# S237. THE ACCEPTANCE, FEASIBILITY AND PRELIMINARY EFFECTS OF DYNAMIC INTERACTIVE SOCIAL COGNITION TRAINING IN VIRTUAL REALITY (DISCOVR): A PILOT STUDY

**DOI:** 10.1093/schbul/sby018.1024

**Published:** 2018-04-01

**Authors:** Saskia Nijman, Wim Veling, Chris Geraets, Marieke Pijnenborg

**Affiliations:** 1 GGZ Drenthe; 2 University Medical Center Groningen; 3 GGZ Drenthe, Rijksuniversiteit Groningen

## Abstract

**Background:**

Many people with psychotic disorder experience problems in social functioning, such as finding and maintaining jobs and relationships, which have been shown to be strongly related to deficits in social cognition. A class of interventions called Social Cognition Training (SCT) aims to improve social cognition through practice and strategy training. SCT has been shown to have positive effects on social cognition. (Social) cognition training, however, is known to optimally translate to functional skills when it is applied to and integrated with different areas of daily life. To promote the transfer of training skills to functional domains, it may therefore be beneficial to provide SCT in virtual reality (VR), since it closely resembles real-life social situations. VR is highly realistic and interactive, allowing for practice of social situations in ecologically valid environments. VR is also controllable, allowing for personalization of situations and difficulty level. In the present study, we tested the acceptance and feasibility of a newly developed VR SCT called ‘DiSCoVR’ (Dynamic Interactive Social Cognition Training in Virtual Reality).

**Methods:**

Twenty-two individuals with a psychotic disorder were recruited from three mental health institutions in the Netherlands. All participants received a VR SCT, which was aimed at three domains: 1) emotion perception (identifying virtual characters’ emotions in a virtual street); 2) social perception and theory of mind (understanding social situations and the thoughts, emotions and behavior of virtual characters); and 3) practicing social interactions with a virtual character. The intervention strongly emphasized practice with social situations in VR between, and with, virtual characters. Participants also learned strategies to cope with difficulties they experienced in social situations. Participants were assessed at baseline and post-treatment. Acceptance of the intervention was evaluated at post-treatment using a questionnaire. Social cognition was also assessed (emotion perception, social perception and theory of mind) using video/photo tasks and stories. Finally, psychotic symptoms, social anxiety, paranoia, self-esteem and depression were measured using an interview and questionnaires.

**Results:**

The results of this pilot study will be presented, focusing on the findings regarding acceptance and feasibility, but also social cognition and other secondary outcome domains.

**Discussion:**

The implications of the findings of the pilot study will be discussed in the context of the preparation of a randomized controlled trial of DiSCoVR (for example, necessary alterations to the protocol and/or VR software). Plans for this randomized controlled trial will be discussed.

